# Activity and safety of apatinib monotherapy or apatinib combined with chemotherapy for patients with metastatic or unresectable osteosarcoma over the age of 40 years: A retrospective analysis

**DOI:** 10.3389/fonc.2022.1031787

**Published:** 2022-11-01

**Authors:** Taojun Gong, Qi Huang, Fan Tang, Yitian Wang, Zhuangzhuang Li, Yi Luo, Li Min, Yong Zhou, Chongqi Tu

**Affiliations:** ^1^Department of Orthopedics, Orthopaedic Research Institute, West China Hospital, Sichuan University, Chengdu, Sichuan, China; ^2^Operating Room, West China Hospital, Sichuan University/West China School of Nursing, Sichuan University, Chengdu, Sichuan, China

**Keywords:** osteosarcoma, apatinib, chemotherapy, metastasis, older patients

## Abstract

**Background:**

Osteosarcoma commonly develops during childhood and adolescence. Only one-third of osteosarcoma patients have been clinically detected over the age of 40 years, and the survivorship of those patients is quite dismal. Apatinib, a novel multitarget angiogenesis inhibitor, has shown a short-term efficacy in advanced or metastatic osteosarcoma. However, the data for apatinib in the older patients with osteosarcoma are limited. We aim to evaluate the efficacy and safety of apatinib combined with chemotherapy versus apatinib monotherapy in the treatment of patients over 40 years old with metastatic or unresectable osteosarcoma.

**Methods:**

We retrospectively analyzed the patients with metastatic osteosarcoma who were treated with apatinib monotherapy or apatinib combined with chemotherapy between May 2015 and December 2018 in the Department of Orthopedics at West China Hospital. Apatinib was initially administered with a dose of 500 mg daily, and the dose was adjusted according to toxicity. The objective response rate (ORR), disease control rate (DCR), duration of response (DOR), progression-free survival (PFS), and overall survival (OS) were investigated. The treatment-related adverse events and the safety of apatinib were also documented.

**Results:**

A total of 45 patients (28 men, 17 women) with metastatic or unresectable osteosarcoma were finally included, and 41 patients received at least one cycle of treatment and were evaluable for efficacy. Of 41 patients, 24 who were intolerant to intensive chemotherapy or have failed standard chemotherapy received apatinib monotherapy, and 17 patients were treated with apatinib plus chemotherapy. The median PFS and median OS were longer in the group treated with apatinib combined with chemotherapy than those of the apatinib monotherapy group (5.6 months *vs*. 2.6 months; 15.1 months *vs*. 9.7 months). Moreover, the median DOR was significantly prolonged in the group treated with apatinib combined with chemotherapy compared with that in the monotherapy group.

**Conclusion:**

Apatinib demonstrated promising activity in patients over 40 years old with metastatic or unresectable osteosarcoma. The combination of apatinib and chemotherapy conferred a durable response compared with apatinib monotherapy, which might be an alternative therapeutic strategy for the management of osteosarcoma in older patients.

## Introduction

Osteosarcoma, the most common primary malignant bone tumor, is characterized by a bimodal age distribution peak in young adolescents and older adults (1). Most patients developed osteosarcoma between the age of 14 and 18 years, and about 13% to 30% of patients developed osteosarcoma at age over 40 years (2). As the geriatric populations in society are rapidly growing, the number of older patients with osteosarcoma has been increasing worldwide (3). However, older patients with osteosarcoma demonstrated distinct clinical characteristics compared with young patients, including high incidence of axial site involvement, frequent metastasis at diagnosis, and delayed diagnosis, different for both host and tumor biology (4). Generally, adult patients older than 40 years face a worse clinical prognosis compared with younger patients (5). Conventional chemotherapy consisting of high-dose methotrexate, doxorubicin, cisplatin, and ifosfamide is recommended as the standard first line of treatment for younger patients with osteosarcomas, while these chemotherapy regimens are not well applicable to older patients due to poor tolerance and response to aggressive chemotherapy and poor treatment compliance (6). Even with the combination of surgical resection and neoadjuvant chemotherapy, the overall survival rate of older adults with non-metastatic disease was approximately 50% (7), whereas for patients with metastasis at diagnosis, the 5-year overall survival rate plummets to less than 15% (5, 8). Currently, treatment strategies for older patients with osteosarcoma vary among institutions. There are no standardized treatment options that exist in terms of the choice of second-line treatment following the first-line chemotherapy (4).

In recent years, small-molecule anti-angiogenesis tyrosine kinase inhibitors (TKIs) as a new nonchemotherapeutic systemic treatment has exhibited promising clinical results in osteosarcoma patients (9). Sorafenib and regorafenib with substantial improvement in PFS have been approved as the agents complementary to the cytotoxic chemotherapy for patients with relapsed or refractory osteosarcoma (10, 11). Apatinib as a specifically targeting VEGFR-2 and oral receptor tyrosine kinase inhibitor has been approved by the Chinese Food and Drug Administration (CFDA) to treat advanced and metastatic gastric cancer in 2014. Regarding the management of osteosarcoma, preclinical studies have shown that apatinib promotes apoptosis and autophagy through VEGFR2/STAT3/BCL-2 signaling pathways in osteosarcoma cells, as well as attenuates invasion and migration through RhoA/ROCK/LIMK2 pathways (12, 13). Furthermore, a phase II prospective clinical trial conducted at Peking University People’s Hospital has revealed that apatinib exhibits an impressive response rate and tolerable safety profile in patients with advanced or metastatic osteosarcoma after the failure of chemotherapy (14). These results indicate that apatinib might be an alternative therapeutic option for patients with osteosarcoma. Nevertheless, the clinical effectiveness of apatinib in previous studies was investigated as the second or later-line treatment in the population mainly constituted by younger patients (14–19). However, the efficacy of apatinib in the treatment for older patients with osteosarcoma has not been reported so far. To provide clinical evidence for real-world practice, we retrospectively evaluated the efficacy and safety of apatinib monotherapy or apatinib combined with chemotherapy in patients over 40 years old with unresectable or metastatic osteosarcoma.

## Patients and methods

### Patient enrolment

The medical records of patients with metastatic osteosarcoma who received apatinib treatment between May 2015 and December 2018 in the Department of Orthopedics at West China Hospital were collected and analyzed. The inclusion criteria were as follows: (1) age ≥40 years; (2) diagnosis of histologically confirmed osteosarcoma; (3) having unresectable or metastatic tumor lesions that were not suitable for curative treatment; (4) administration of apatinib as a subsequent line treatment in patients with progression after standard chemotherapy or patients who were intolerant to or refused chemotherapy; (5) at least a measurable lesion according to the Response Evaluation Criteria in Solid Tumors (RECIST 1.1); (6) an eastern cooperative oncology group performance (ECOG) status of 0–2; and (7) adequate renal, hepatic, and hematopoietic function of the patient. Patients who had previously received other targeted therapies or had a concomitant malignant tumor, cardiac insufficiency or arrhythmia, or a metastatic lesion that developed in the brain were excluded. Written informed consent for off-label treatment was collected from all patients before apatinib treatment. This study was conducted according to the principles described in the Declaration of Helsinki and approved by the Institutional Review Board of Sichuan University West China Hospital.

### Treatment methods

The starting dose of apatinib was orally administrated with 500 mg once daily, and a duration of 28 days was defined as one treatment cycle. During the treatment, the adverse events were monitored, and the dosage of apatinib was adjusted according to hematological or non-hematological toxicity. Adverse events that occurred during apatinib treatment were managed with symptomatic treatment, dosage adjustment, or treatment termination. When a patient experienced any grade 3 or 4 adverse events, the apatinib treatment was interrupted temporarily until the symptom was relieved. If the toxicity-related events were adequately remitted after 2 weeks of drug interruption, the apatinib treatment was reinstituted with the original dosage. In contrast, the dosage was reduced by a stepwise strategy: the first dosage adjustment was 425 mg once daily; the second dosage adjustment was 250 mg once daily. If the adverse events did not resolve by the dosage adjustment of 250 mg, the treatment of apatinib was terminated.

### Response assessment

The last date for the assessment of clinical outcomes was May 31, 2021. All patients who received apatinib treatment were included in the safety and toxicity assessment, and only those who had received at least one cycle of apatinib were evaluable for efficacy. The baseline assessment included chest computed tomography evaluation of the pulmonary lesions and magnetic resonance imaging for musculoskeletal lesions. Bone scintigraphy was used to assess the distant bone metastases of patients at the baseline and detect new lesions of bone metastases during the treatment. Treatment efficacy was assessed after two treatment cycles or more frequently in patients with substantial progression or discontinuous treatment. Tumor response was defined as a complete response (CR), partial response (PR), stable disease (SD), or progressive disease (PD) according to the RECIST 1.1 criteria (20). The primary endpoint was progression-free survival (PFS) and identified as the time from the initiation date of apatinib administration to the date of disease progression. Overall survival (OS) referred to the duration from treatment initiation to death from any cause or the last known follow-up date. Disease control rate (DCR) was defined as the percentage of patients with CR, PR, and SD on record. The objective response rate (ORR) was defined as the percentage of patients who achieved CR and PR. Duration of response (DOR) was defined as the time from the first documentation of objective response to the time of the first documentation of disease progression or death, whichever came first. Drug-related adverse events were evaluated and graded by using the National Cancer Institute Common Terminology Criteria for Adverse Events (version 4.0).

### Statistical analysis

All statistical analysis was performed using the STATA Statistical Software (version 16, College Station, TX, USA). Descriptive statistics of mean and standard deviation or range was used for continuous variables and categorical variables (number and percentage). The chi-square test (or Fisher’s exact test) was used for the analysis of categorical variables, and the Wilcoxon rank sum test was used for continuous variables. The Kaplan–Meier curves were drawn to compare the difference in PFS and OS between groups, and the survival differences were compared using the log-rank test. A two-tailed p value <0.05 was considered statistically significant.

## Results

### Patients’ characteristics

**Figure 1:**
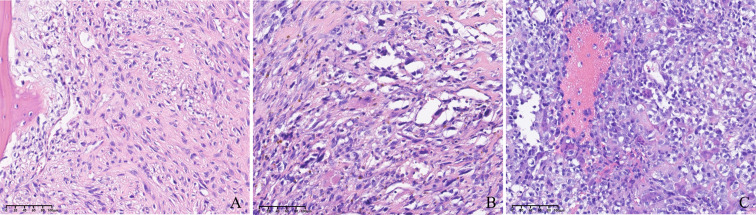
**(A)** Image of conventional osteosarcoma hematoxylin and eosin (H&E) staining (200×). **(B)** Image of telangiectatic osteosarcoma H&E staining (200×). **(C)** Image of small-cell osteosarcoma H&E staining (200×).

From May 2015 to December 2018, 45 patients (28 men, 17 women) with unresectable or metastatic osteosarcoma treated with apatinib were retrospectively identified. The demographics and clinical baseline characteristics of patients before apatinib treatment are shown in **Table 1**. The median age was 49 (range 40–88) years, including 28 men and 17 women with a male-to-female ratio of 1.6:1. ECOG score 0–1 was noted in 29 patients (64.4%), and score 2 in 16 patients (35.5%). The primary tumor location was the extremity in 40 patients (88.9%), spine in two patients (4.4%), and pelvis in three patients (6.7%). Histologically, the major histologic subtype was primary osteosarcoma in 45 patients, including 38 patients with conventional (84.4%), five patients with telangiectatic (11.1%), and two patients with small cell osteosarcoma (4.2%). The typical images of three types of osteosarcoma hematoxylin and eosin staining are showed in **Figures 1A–C**. Previously surgical treatment for primary lesions was observed in 35 patients (77.8%) (33 patients reached a surgical margin of R0, two patients reached the R1 margin, and no patient received an R2 surgical margin), while the remaining 10 patients (22.2%) did not receive surgery because of insufficient systemic conditions (six patients) or inoperative local conditions (four patients). The sites of distant metastases included lung only in 30 patients (66.7%), bone only in five patients (11.1%), and both bone and lung in six patients (13.3%). Four patients (8.9%) had initially unresectable lesions; 18 (40.0%) of the enrolled patients who had failed first-line chemotherapy and 27 (60.0%) patients who received failed second-line chemotherapy selected apatinib as the subsequent treatment. The current regimens were apatinib monotherapy in 25 patients (55.6%). The combined chemotherapy included high-dose methotrexate (8–12 g/m^2^, days 3 and 4), doxorubicin (60–80 mg/m^2^, days 1 and 2), and cisplatin (100–120 mg/m^2^, day 1) (MAP) in eight patients (17.8%) (6–10 circles); doxorubicin (60–80 mg/m^2^, days 1 and 2) and cisplatin (100–120 mg/m^2^, day 1) (AP) in 10 patients (22.6%) (8–12 circles); and MAP plus ifosfamide (2 g/m^2^·d, day 5–10) in two patients (4.4%) (eight and nine circles, respectively).

**Table 1:** Clinical baseline characteristics of the patients with metastatic osteosarcoma.

Characteristics	Total	AM (n = 25)			AC (n = 20)
**Age**					
Median age (year)	49	49			48
Range	40–88	40–72			42–88
**Gender**					
Male	28 (62.2%)	14 (31.1%)			14 (31.1%)
Female	17 (37.8%)	11 (24.4%)			6 (13.4%)
**ECOG performance status**					
0-1	29 (64.4%)	14 (31.1%)			15 (33.3%)
2	16 (35.5%)	11 (24.4%)			5 (11.1%)
**Primary tumor location**					
Extremity	40 (88.9%)	23 (51.1%)			17 (37.8%)
Spine	2 (4.4%)	1 (2.2%)			1 (2.2%)
Pelvis	3 (6.7%)	1 (2.2%)			2 (4.5%)
**Histologic subtypes**					
Conventional ^a^	38 (84.4%)	22 (48.9%)			16 (35.5%)
Telangiectatic	5 (11.1%)	2 (4.4%)			3 (6.7%)
Small cell	2 (4.5 %)	1 (2.2%)			1 (2.2%)
**Excision of primary lesion**					
Yes	35 (77.8%)	22 (48.9%)			13 (28.9%)
No	10 (22.2%)	3 (6.7%)			7 (15.6%)
**Distant metastases**					
Lung only	30 (66.7%)	13 (28.9%)			17 (37.8%)
Bone only	5 (11.1%)	5 (11.1%)			0
Both bone and lung	6 (13.3%)	5 (11.1%)			1 (2.2%)
Unresectable lesion	4 (8.9%)	2 (4.4%)			2 (4.4%)
**Previous chemotherapy ^b^**					
First-line chemotherapy	18 (40.0%)	11 (24.4%)			7 (15.6%)
Second-line chemotherapy	27 (60.0%)	14 (31.1%)			13 (28.9%)
**Previous radiotherapy**					
Yes	3 (6.7%)	1 (2.2%)			2 (2.2%)
No	42 (93.3%)	24 (53.3%)			18 (40.0%)
**Current treatment strategy**					
Apatinib monotherapy	25 (55.6%)	25 (55.6%)			–
Apatinib + MAP	8 (17.8%)	–			8 (17.8%)
Apatinib + AP	10 (22.2%)	–			10 (22.2%)
Apatinib + MAP and ifosfamide	2 (4.4%)	–			2 (4.4%)

AM, apatinib monotherapy; AC, apatinib combined with chemotherapy; ECOG, Eastern Cooperative Oncology Group; M, methotrexate; A, doxorubicin; P, cisplatin; I, ifosfamide.

^a,^ Conventional type including osteoblastic, fibroblastic, chondroblstic osteosarcoma.

^b,^ First-line chemotherapy was defined as high-dose methotrexate, doxorubicin, cisplatin, and ifosfamide.

### Treatment outcomes

**Figure 2:**
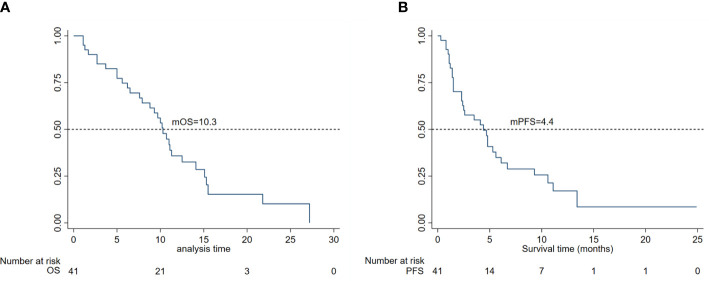
**(A)** Kaplan–Meier curves for overall survival and **(B)** progression-free survival in 45 patients.

In total, 41 of the 45 patients with unresectable or metastatic osteosarcoma treated with apatinib were evaluable for efficacy, and the remaining four patients were only assessed for safety due to receiving less than one cycle of treatment. The median duration of medication was 4.8 (95% CI 3.5–7.9) months. The best responses according to RECIST 1.1 at the final evaluation included no patient with CR, 10 patients with PR, 22 patients with SD, and nine patients with PD, giving a DCR of 76.2% (32/41) and ORR of 23.8% (10/41). The estimated median OS (mOS) of the 41 patients was 10.3 months (95% CI, 7.6–12.5) and median PFS (mPFS) was 4.4 months (95% CI, 2.3–6.1), as shown in **Figure 2**.

According to different therapeutic strategies of apatinib treatment, patients were retrospectively divided into two groups, namely the apatinib monotherapy group (AM) and the apatinib in combination with chemotherapy treatment group (AC). For the patients in the AM group, ORR was 8.3% (2/24) and DCR was 79.2% (19/24), including two patients who achieved PR and 17 patients who obtained SD. For patients treated with the combination of apatinib and chemotherapy, the ORR was 47.0% with eight patients achieving PR, and DCR was 88.2% with seven patients reaching SD, as displaced in **Table 2**.

**Table 2:** Best response of patients during apatinib treatment according to different groups.

Best response	Apatinib monotherapy (n = 24)	Apatinib combine with chemotherapy (n = 17)
CR	0	0
PR	2	8
SD	19	7
PD	3	2
ORR	8.3%	47.0%
DCR	79.2%	88.2%

PR, partial response; SD, stable disease; PD, progressive disease.

### Subgroup analysis

**Figure 3:**
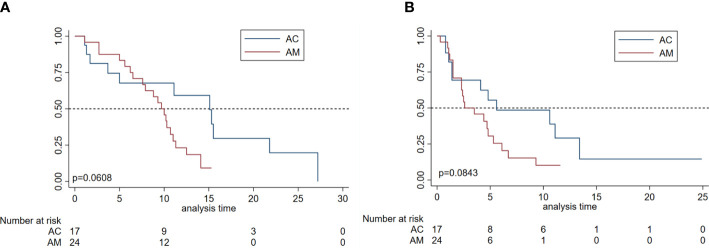
Survival analyses by subgroup in patients with metastatic osteosarcoma. **(A)** Overall survival and progression-free survival **(B)** of patients according to the apatinib monotherapy group (AM) and apatinib plus chemotherapy treatment group (AC).

Regarding the patients in the different subgroups, the mPFS of patients treated with AM and AC was 2.6 (95% CI, 1.5–4.8) and 5.6 (95% CI, 1.4–13.4) months, respectively; the mOS of patients treated with AM and AC was 9.7 (95% CI, 6.5–11.0) and 15.1 (95% CI, 3.7–21.8) months, respectively. However, there was no significant difference in mOS (p = 0.0608) and mPFS for both groups (p = 0.0843), as shown in **Figure 3**. Of note, a trend was observed in which the patients treated with the combination of apatinib and chemotherapy were more likely to have a longer mPFS and mOS than those of patients treated with monotherapy. Furthermore, the median DOR of patients treated with AC and AM was 11.0 months (95% CI, 1.7–NA) and 3.6 months (95% CI, 2.6–5.3), respectively. Patients treated with the combination of apatinib and chemotherapy obtained a longer-term response than those patients who were treated with monotherapy (p = 0.057).

### Safety and toxicity

Safety analyses were based on the 45 eligible patients, including four patients who received less than one cycle of treatment owing to personal refusal. The overall incidence of apatinib-related adverse events was 84.3%, and most drug-related adverse events that occurred during treatment were mild (grades 1–2) and controllable. None of the patients discontinued treatment because of uncontrolled adverse events, whereas seven patients (13.7%) (five were treated with apatinib combined with chemotherapy, whereas two received apatinib alone) had to adjust the dose to 425 mg once daily, and three patients (5.8%) (two were treated with apatinib combined with chemotherapy and one received apatinib alone) had to adjust the dose to 250 mg once daily because of the intolerable drug-related toxicity including hand–foot syndrome, hypertension, pneumothorax, and diarrhea. All of these grade-3 adverse events were effectively relieved after dose reduction and symptomatic treatment. The overall incidence of grade-3 adverse events was 23.5%, but there was no statistically significant difference between any two groups. No drug-related deaths were observed during treatment, and all deaths were attributed to disease progression. The common adverse events included hand–foot skin syndrome (n = 16, 35.6%), hypertension (n = 15, 33.3%), fatigue (n = 13, 28.9%), and pneumothorax (n = 9, 20.0%). For hematological toxic effects, the combination of apatinib and chemotherapy numerically increased the rate of grade 2 or 3 adverse events, whereas no significant difference was found between any two groups for the rate of nonhematological toxic effects (p = 0.062). The detailed drug-related adverse events are summarized in **Table 3**.

**Table 3:** Treatment-related adverse events that occurred during apatinib treatment.

Adverse events	Total, n (%)	Apatinib monotherapy (n = 25)			Apatinib + chemotherapy (n = 20)		
		Grade 1	Grade 2	Grade 3–4	Grade 1	Grade 2	Grade 3–4
Hand–foot syndrome	16 (35.6%)	4	3	1	3	3	2
Hypertension	15 (33.3%)	3	2	2	4	3	1
Fatigue	13 (28.9%)	4	1	0	6	2	0
Pneumothorax	9 (20.0%)	1	2	1	2	3	0
Neutropenia	9 (20.0%)	1	0	0	2	4	2
Proteinuria	8 (17.8%)	2	1	0	4	1	0
Diarrhea	8 (17.8%)	1	2	1	2	1	1
Anemia	7 (15.6%)	1	1	0	3	2	0
Hair hypopigmentation	6 (13.3%)	1	2	0	1	2	0
Oral mucositis	6 (13.3%)	2	1	0	2	1	0
Vomiting	6 (13.3%)	1	0	1	2	2	0
Thrombocytopenia	5 (11.1%)	1	0	0	2	2	0
Skin hypopigmentation	5 (11.1%)	2	0	0	2	1	0
Abdominal distention	5 (11.1%)	1	1	0	2	1	0
Myalgia/arthralgia	5 (11.1%)	1	1	0	1	2	0
Apositia	4 (8.9%)	1	1	0	1	1	0
Wound dehiscence	3 (6.7%)	1	0	0	2	0	0
Rash acneiform	1 (2.2%)	0	0	0	1	0	0
Elevated aminotransferase	1 (2.2%)	0	1	0	0	0	0

## Discussion

Despite the considerable improvements of multimodal therapy for osteosarcoma, the survivorship of patients over 40 years old with metastatic or unresectable osteosarcoma has remained stagnant during the last four decades (21). In recent years, anti-angiogenesis tyrosine kinase inhibitors have contributed an important option to manage metastatic osteosarcoma, particularly for patients who have disease progression after chemotherapy failure (14, 17).

Previous literature demonstrated that older osteosarcoma patients suffered a worse clinical outcome when compared with young patients (22). The 5-year overall survival rates of osteosarcoma patients aged over 40 years without metastasis at diagnosis were 38.5%, whereas it slumped to 2.5% in patients who developed distant metastasis with a median overall survival of 7.0 months (23). In general, due to concomitant comorbidities with generally poor health, many elderly patients may not be tolerant of a high-dose and intensive chemotherapy, especially in the setting of metastatic disease (8). Moreover, the efficacy of chemotherapy in the treatment of osteosarcoma patients aged over 40 years remains controversial (23–26). Adjuvant chemotherapy did not improve the survival of osteosarcoma patients aged over 40 years unlike in patients younger than 40 years (23, 24). Additionally, radiotherapy predicted a worse prognosis of older patients with osteosarcoma, indicating that radiotherapy was not an appropriate strategy for these patients (23). In our study, of 45 patients aged over 40 years, 27 patients received apatinib monotherapy and 18 patients were administrated with apatinib plus chemotherapy. The administration of apatinib induced tumor shrinkage in two patients with ORR of 8.3% and DCR of 79.2%. The mOS of those patients was 9.7 months, which was slightly prolonged compared with the data previously reported (23), but lower than that of patients in the apatinib combined with chemotherapy treatment group. The possible explanations for this result might be that a portion of patients (4/24) who received apatinib monotherapy did not undergo surgical treatment, both of which were correlated with a poor prognosis of osteosarcoma patients (22). Furthermore, in the apatinib combined with chemotherapy treatment group, we observed a higher ORR and DCR (47.0% and 88.2%, respectively) and a longer mOS and mPFS (15.1 and 5.6 months, respectively), which indicated that apatinib and chemotherapy have a synergistic effect in treating older patients with osteosarcoma. During the treatment, the common adverse events associated with apatinib were acceptable, and there were no grade 4 adverse events, Meanwhile, seven patients experienced grade 3 adverse events, which were remitted after dose reduction. Thus, apatinib is relatively tolerable for older patients in comparison with the chemotherapy-related toxicity, such as peripheral neuropathy, hematological toxicity, and nephrotoxicity.

For the second-line treatment for metastatic osteosarcoma after the failure of chemotherapy, apatinib has shown definite antitumor activity with a high response rate and tolerable toxicity profile (14–19) (**Table 4**). In a phase II, open-label clinical trial, 37 patients with advanced or metastatic osteosarcoma after the failure of standard chemotherapy received 500 or 750 mg of apatinib treatment until an unacceptable toxicity or disease progression. The results described that the median PFS and OS were 4.5 months and 9.87 months, respectively (14). The partial response rate was 43.24% in this trial, which was comparable with that reported in regorafenib and sorafenib (10, 11). In our study, patients with metastatic or unresectable osteosarcoma who had disease progression after chemotherapy failure received the apatinib monotherapy or apatinib combined with chemotherapy as the subsequent therapy. Regarding the clinical outcomes of patients, mPFS was 4.4 months, and mOS was 10.3 months with an ORR of 23.8%, which were basically consistent with the results of other retrospective studies (15–19). The ORR was worse than that reported by Xie et al. (14); we considered that drug dose essentially affects drug efficacy. Some patients were administrated 750 mg, which was higher than the dosage in our study. In addition, the median age was 19 (range 16–62) in the prospective clinical trial; younger patients may have responded better than older patients in our study. Although apatinib demonstrated meaningful efficacy in metastatic osteosarcoma, it slightly improved the survival values similar to the studies involving other TKIs (10, 11, 14, 27). Furthermore, the treatment response duration of apatinib therapy was less than 6 months with a median DOR of 5.07 months, suggesting that the antitumor activity of apatinib was short term (14). In particular, when patients were treated with apatinib as the first-line treatment before the chemotherapy, the DOR was only 3.45 months.

**Table 4:** Recent clinical studies of apatinib treatment for advanced or metastatic osteosarcoma.

Author	Year	Case	Age (range)	Inclusion criteria	Initial dose of apatinib	DCR	ORR	mPFS (M)	mOS (M)
Xie	2018	37	19 (16–62)	Unresectable; advanced; metastatic osteosarcoma after failure of chemotherapy	750 mg for body surface area ≥1.5;	NA	43.24	4.5	9.8
					500 mg daily for BSA <1.5				
Zheng	2018	10	16 (12–30)	Advanced; metastatic osteosarcoma failure of chemotherapy	500 mg for adults;	70%	20%	7.5	14
					250 mg for children				
Tian	2019	27	20.8 (NA)	Metastatic osteosarcoma failure of chemotherapy	750 mg for body surface area ≥1.5;	66.67%	25.93%	3.5	9.5
					500 mg daily for BSA <1.5				
Liu	2020	105	33 (18–58)	Metastatic osteosarcoma failure of chemotherapy	750 mg for body surface area ≥1.5;	77.14%	37.14%	4.1	9
					500 mg daily for BSA <1.5				
Tian	2020	19	22.4 (NA)	Metastatic osteosarcoma	500 mg	63.10%	15.70%	4.67	NA
Liao	2020	18	NA	Metastatic osteosarcoma failure of chemotherapy	500 mg	78.79%	6.06%	7.89	17.61
Present study		45	49 (40–88)	Age 40 and older; metastatic; unresectable osteosarcoma	500mg and dosage adjustment by AEs	76.2%	23.8%	4.4	10.3

mPFS, median progression-free survival; mOS, median overall survival; ORR, objective response rate; DCR, disease control rate; M, months; NA, not achieved; BSA, body surface area; AEs, adverse events.

Recently, the combination of anti-angiogenesis and chemotherapy has shown superior clinical efficacy than monotherapy in several cancer types. Accordingly, the combination of chemotherapy was associated with more grade 3 or 4 hematological toxic effects than TKI monotherapy (28). A meta-analysis enrolling 19 studies with 1256 gastric cancer patients concluded that the combination of apatinib and chemotherapy was associated with a favorable efficacy when compared with chemotherapy alone, but with a high rate of drug-related toxicity (29). In our study, 40.0% (18/45) of patients with metastatic osteosarcoma received a combination of apatinib and chemotherapy. Importantly, we note that the mPFS and mOS of the combined treatment group were longer than those of the monotherapy group (5.6 months *vs*. 2.6 months; 15.1 months *vs*. 9.7 months), although the difference was not statistically significant.

*In vitro*, apatinib was able to reverse the multidrug resistance in cancer cells by inhibiting multidrug resistance protein 1 (MRP1) and breast cancer resistance protein (BCRP)-mediated drug transport function (30). Tang revealed that apatinib sensitizes triple-negative breast cancer cells to doxorubicin *in vitro* and *in vivo* through the inactivation of NF-κB signaling pathways (31). Moreover, apatinib was demonstrated to enhance the sensitivity of osteosarcoma cells to doxorubicin and inhibited the doxorubicin-induced stemness phenotype through STAT3/Sox2 pathway inactivation (32). Thus, this result could be attributed to the sensitizing activity of apatinib that combated the chemoresistance induced by doxorubicin and the inhibition of VEGF-mediated angiogenesis. We hypothesized that the absence of a statistically significant difference might be attributed to the limited sample size and heterogeneity between the different groups in a real-world setting. Moreover, the median DOR of the combined treatment group was 11.0 months, which was significantly longer than that of the monotherapy treatment group (3.6 months), indicating that the combination with chemotherapy exhibited a synergistic effect and might extend the therapeutic effect of apatinib. Regarding safety, the combination of apatinib and chemotherapy numerically increased the rate of grade 2 or 3 hematological toxic effects. Since chemotherapy itself could also induce severe hematological adverse events, we sometimes hardly distinguish whether the drug-related toxicity was related to TKIs or chemotherapy. For the rate of non-hematological toxic effects, no significant difference was found between any two groups.

Although the preliminary conclusion was drawn from our results, limitations inevitably existed in this study. As a retrospective study, the sample size was very limited: only 45 patients were included. The heterogeneity of the patient population and the small cohort size of patients in subgroups might further influence the accuracy of the results. Furthermore, most of the cases in this study were at ECOG 0 or 1 severity, probably because patients with a poor general condition cannot tolerate the side effects from anti-angiogenesis therapy or chemotherapy and other preferred supportive treatments. The smaller proportion of ECOG 2 patients might have affected the precise analysis survival of patients. Apart from that, the patients were retrospectively enrolled in this study, which might be prone to recall bias and confounding.

## Conclusion

In conclusion, apatinib demonstrated encouraging activity in older patients with metastatic or unresectable osteosarcoma and significantly delayed disease progression. In a real-world experience, apatinib might have an important therapeutic role as an agent complementary to standard cytotoxic chemotherapy against osteosarcoma, especially for an older patient who is intolerant or insensitive to intensive chemotherapy. Furthermore, the combination with chemotherapy leads to a better survival benefit and longer response duration than monotherapy, which might be an optional therapeutic strategy for the management of osteosarcoma. Prospective and randomized trials with a large sample size to validate this new combination strategy are warranted.

## Data availability statement

The original contributions presented in the study are included in the article/supplementary material. Further inquiries can be directed to the corresponding authors.

## Ethics statement

The studies involving human participants were reviewed and approved by Biological and Medical Ethics Committee of West China Hospital. The patients/participants provided their written informed consent to participate in this study.

## Author contributions

TG and QH contributed equally to this work and are co-first authors. TG analyzed and interpreted the data. TG and QH wrote and edited the manuscript. FT, YW, ZL, YL, and LM reviewed the manuscript. YZ and CT supervised and reviewed the manuscript. All authors contributed to the article and approved the submitted version.

## Funding

This work was supported by the 1.3.5 project for disciplines of excellence, West China Hospital, Sichuan University (No. ZYJC18036).

## Conflict of interest

The authors declare that the research was conducted in the absence of any commercial or financial relationships that could be construed as a potential conflict of interest.

## Publisher’s note

All claims expressed in this article are solely those of the authors and do not necessarily represent those of their affiliated organizations, or those of the publisher, the editors and the reviewers. Any product that may be evaluated in this article, or claim that may be made by its manufacturer, is not guaranteed or endorsed by the publisher.
